# Uncompromised Treatment Efficacy in Elderly Patients With Hepatocellular Carcinoma: A Propensity Score Analysis

**DOI:** 10.1097/MD.0000000000000264

**Published:** 2014-12-02

**Authors:** Po-Hong Liu, Chia-Yang Hsu, Yun-Hsuan Lee, Cheng-Yuan Hsia, Yi-Hsiang Huang, Chien-Wei Su, Yi-You Chiou, Han-Chieh Lin, Teh-Ia Huo

**Affiliations:** From the Faculty of Medicine (PHL, CYH, YHL, CYH, CWS, YYC, HCL, TIH); Institute of Clinical Medicine (YHH); Institute of Pharmacology (TIH), National Yang-Ming University School of Medicine, Taipei, Taiwan; Department of Medicine (PHL, CYH, YHL, YHH, CWS, HCL, TIH); Department of Surgery (CYH); Department of Radiology, Taipei Veterans General Hospital, Taipei, Taiwan (YYC); and Department of Biostatistics, UCLA, Los Angeles, CA, USA (CYH).

## Abstract

The number of elderly hepatocellular carcinoma (HCC) patients is expected to rise. We analyzed the impact of age on clinical presentations, treatment allocation, and long-term survival between elderly (≥75 years) and younger (<75 years) HCC patients.

In this study, a total of 812 elderly and 2270 younger HCC patients were evaluated. The baseline information and long-term survival were compared in the entire population and in different treatment groups. A propensity score matching analysis with preset caliper width was utilized to compare survival differences in different patient subgroups.

Elderly HCC patients had discrete characteristics compared with younger HCC patients. Elderly patients received surgical resection (SR) less frequently, while more elderly patients underwent radiofrequency ablation (RFA) and transarterial chemoembolization (TACE). Younger patients had significantly better long-term survival than the elderly patients in all patients and in patients receiving SR (both *P* < 0.05). However, of the entire cohort, age was not an independent predictor of poor prognosis in the Cox multivariate model. The long-term survival was similar between 2 age groups in patients receiving RFA and TACE. In the propensity model, there were no significant survival differences among patients receiving SR, RFA, or TACE (all *P* > 0.05). Among the elderly, the Cancer of the Liver Italian Program (CLIP) score provided the lowest Akaike information criterion value.

In conclusion, advanced age is not associated with inferior treatment result in HCC patients receiving different therapeutic modalities. Elderly HCC patients should be encouraged for active treatment when indicated. The CLIP is an optimal prognostic model for outcome assessment.

## INTRODUCTION

The aging society is a global phenomenon. In Taiwan, the average life expectancy at birth is among the longest in the world, being 76 years for males and 83 years for females.^1^ Nearly 12% of total population is more than 65 years old, and more than 5% of population is over 75 years old in Taiwan.^2^ The increase in age causes changes in disease patterns as well as treatment and prognosis.

With an age-standardized incidence rate more than 10/100,000 per year, hepatocellular carcinoma (HCC) is one of the most common malignancies worldwide, accounting for more than 700,000 deaths annually.^3^ In Taiwan, the age-standardized incidence rate was exceedingly high, reaching 32.1/100,000 per year.^4^ HCC is generally diagnosed in middle-aged and elderly individuals.^5^ There is marked geographical variability on the incidence and age distribution of HCC in various countries.^6,7^ Despite different etiological factors, the number of newly diagnosed HCC in the elderly is expected to increase in many parts of the world.^8^ However, the management of HCC of the elderly still poses significant challenges to clinicians due to increased comorbidities, higher incidence of complications associated with treatment, and perceived minimal survival advantages in the elderly.^9–11^

Various studies examined the effectiveness and safety of surgical resection (SR), radiofrequency ablation (RFA), and transarterial chemoembolization (TACE) in elderly patients.^12–14^ Some studies found that younger patients had better prognosis, while others suggested that treatment outcomes were not adversely affected by age.^15,16^ Another report shows that younger HCC patients presented with more aggressive tumor behavior, and were associated with poorer prognosis.^17^ The discrepancy between these studies may be due to diverse demographic features of the enrolled patients. Moreover, unintentional selection bias might develop when elderly patients with better baseline status were included to receive aggressive treatment.

Whether age plays a pivotal role in the assessment and treatment strategy of HCC is under intense debate. This study aimed to investigate the impact of age on treatment allocation and long-term survival up to 10 years in a large patient cohort. A propensity score matching analysis was used to overcome potential confounders and confirm the prognostic impact of old age.

## PATIENTS AND METHODS

### Patients

We analyzed 3082 newly diagnosed HCC patients in Taipei Veterans General Hospital from 2002 to 2013. Comprehensive baseline information, including patient demographics, etiology of liver disease, performance status, tumor characteristics, serum biochemistry, and severity of cirrhosis, was recorded at the time of diagnosis. The survival was inspected every 3 to 4 months until death or dropout from the program. This study was approved by the Institutional Review Board (IRB) of Taipei Veterans General Hospital and complies with the standards of the Declaration of Helsinki. Waiver of patient consent was obtained from the IRB due to retrospective nature of the study, and patient records/information was anonymized and de-identified prior to analysis.

Patients were classified as having elderly HCC if aged ≥75 years. Patients less than 75 years old were classified as younger HCC. This cut-off value was chosen to reflect the aging society in Taiwan and to allow comparison with other relevant studies.^5,15,18^

### Diagnosis and Definitions

The diagnosis of HCC was histologically confirmed or based on the findings of typical radiological features in a 4-phase contrast-enhanced computed tomography scan or dynamic magnetic resonance imaging.^19^ Alcoholism was diagnosed in patients with consumption of alcohol at least 40 g daily for 5 years or more.^20^ The Child–Turcotte–Pugh (CTP) classification was used to define severity of cirrhosis. Total tumor volume (TTV) was calculated as the sum of all the tumor nodule volume.^21^ Macroscopic vascular invasion was defined by presence of adjacent thrombi to the tumor in portal vein with blurring boundary.^22^ Performance status was assessed at the time of diagnosis by the ECOG performance scale.^23^ The Barcelona Clinic Liver Cancer (BCLC), Cancer of the Liver Italian Program (CLIP) classification, Japan Integrated Scoring (JIS) System, and Taipei Integrated Scoring (TIS) System were used to define cancer staging.^21,24–26^

### Treatment

SR was offered to patients with tumor(s) involving no more than 3 Couinaud segments and without main portal vein trunk involvement or distant metastases.^27^ For patients with suitable tumor size and location, RFA was administered using the standard procedure.^28^ TACE was performed in patients who were not eligible or unwilling to receive SR or RFA, and with adequate liver functional reserve and no signs of distant metastases or main portal trunk thrombosis.^22^ Liver transplantation recipients were not included in the current study. Percutaneous ethanol injection, targeted therapy, systemic chemotherapy, and best supportive care were categorized as other treatments. Patients were categorized into treatment groups according to the first active treatment they received. Written informed consent was obtained prior to each treatment.

### Propensity Score Matching Analysis

A propensity score matching analysis was used to generate matched pairs of patients to compare the long-term survival in an observational, non-randomized study.^29,30^ Binary logistic regression with variables associated with age was used to generate propensity scores from 0 to 1. A one-to-one nearest-neighbor match between elderly and younger HCC was used to select patients into subsequent analyses.^31^ Possible variables associated with survival, including age, sex, tumor burden, severity of cirrhosis, vascular invasion, renal function, serum AFP, and diabetes mellitus were included comprehensively for propensity score generation.

### Statistics

The Mann–Whitney *U* test was used to compare continuous variables between 2 groups. The χ^2^ test and 2-tailed Fisher exact test were used to compare categorical data. Kaplan–Meier method with log-rank test was utilized to compare the long-term survival distribution. Prognostic factors that were possibly linked to survival, including gender, etiology of liver disease, severity of cirrhosis, performance status, and tumor extent were included in analysis. After stratification by treatment modalities, factors that were significant in the univariate analysis were introduced into the Cox proportional hazards model to determine the adjusted hazard ratios (HR) and 95% confidence intervals (CI). The predictive accuracy of BCLC, CLIP, JIS, and TIS staging system was compared. Homogeneity was measured by likelihood ratio χ^2^ generated by the Cox model. Akaike information criterion (AIC) was calculated to reveal how the staging systems correlated with patient survival.^32^ A *P*-value less than 0.05 was considered statistically significant. All statistical analyses were conducted with SPSS for Windows version 19 (IBM, Armonk, NY).

## RESULTS

### Patient Characteristics

Patients were categorized as elderly HCC if aged ≥75 years in this study. Eight hundred twelve (27.4%) patients fulfilled this criterion, and 2270 (73.6%) patients were classified as having younger HCC (Table 1). Elderly HCC was significantly associated with a lower prevalence of hepatitis B and alcoholism and a higher prevalence of hepatitis C (all *P* < 0.01). Elderly HCC patients were also associated with lower serum α-fetoprotein (AFP) level, poorer performance status, more advanced BCLC classification (all *P* < 0.001), but lower CLIP score (*P* = 0.006). There were no significant differences in sex, number of tumor(s), CTP score, and TTV between elderly and younger HCC. The allocation of treatment strategy was significantly different. There were 74% of younger HCC patients received curative treatment or TACE as the initial treatment, whereas 67% of elderly HCC patients did so (*P* = 0.001). Elderly patients underwent SR less frequently, and more elderly patients received RFA or TACE (all *P* < 0.05).

**TABLE 1:**
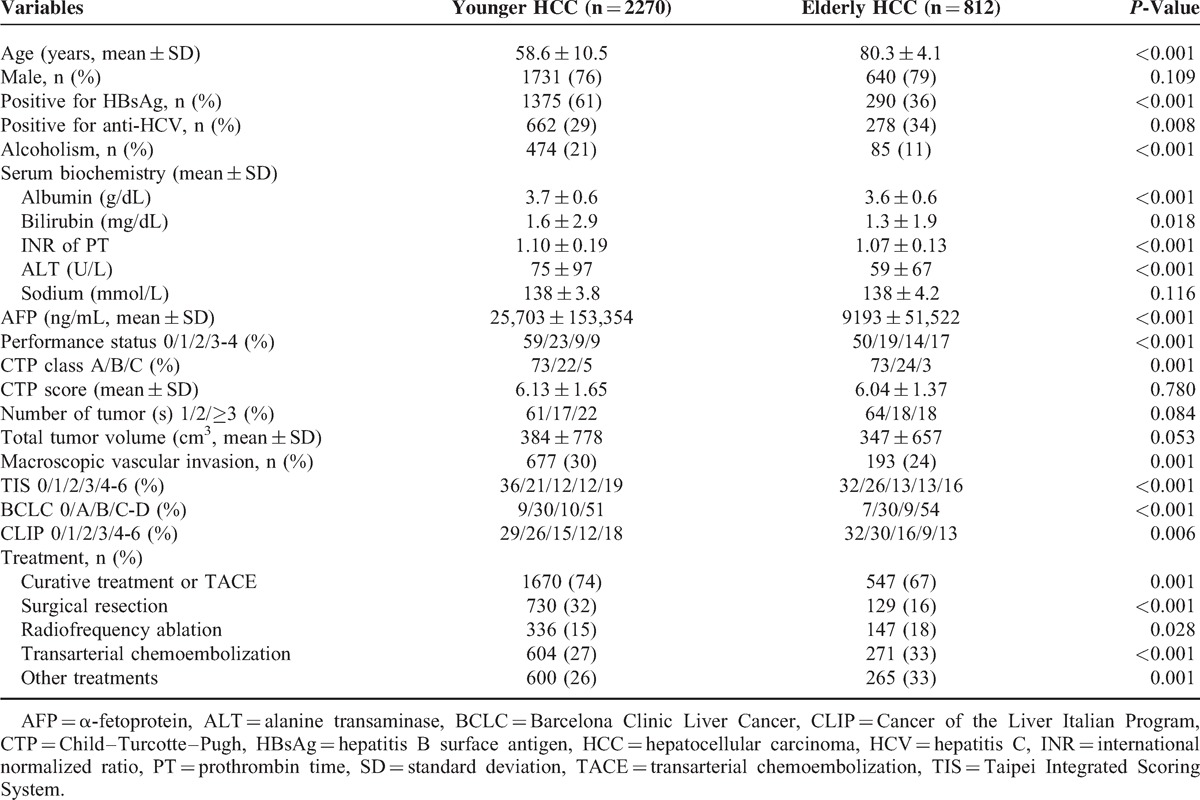
Baseline Demographics in Younger and Elderly Patients With Hepatocellular Carcinoma

**FIGURE 1:**
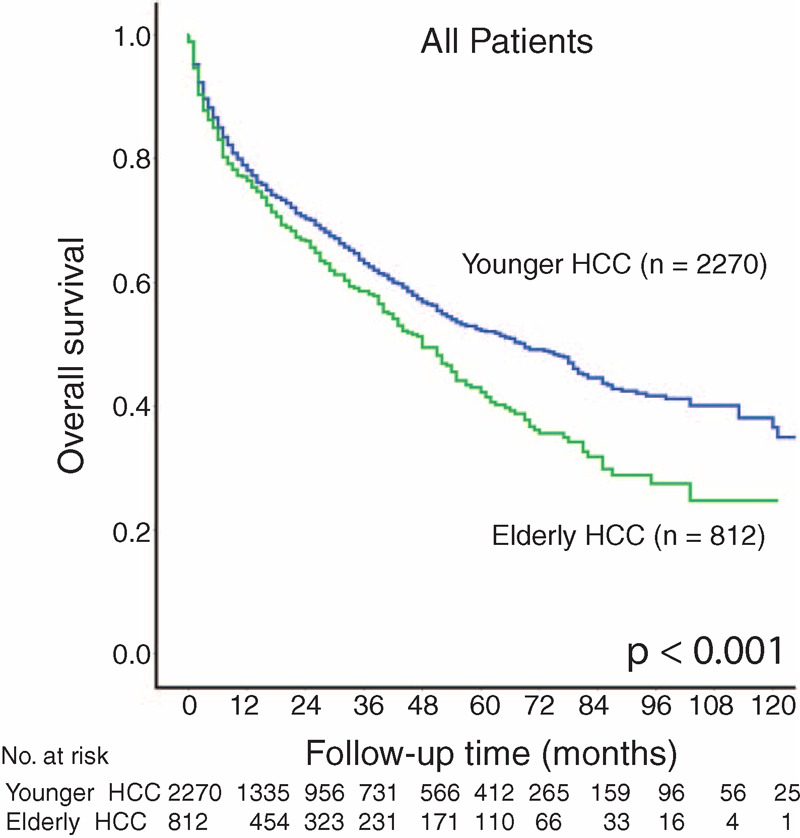
Kaplan–Meier survival plot for elderly and younger hepatocellular carcinoma (HCC) patients.

After a mean follow-up of 28 months, 809 (36%) patients with younger HCC and 337 (42%) patients with elderly HCC died. Younger HCC patients were associated with a significantly better long-term survival (*P* < 0.001, Figure 1); the 1-, 3-, and 5-year survival in younger HCC and elderly HCC were 79% versus 77%, 64% versus 58%, and 52% versus 43%, respectively.

### Surgical Resection

**FIGURE 2:**
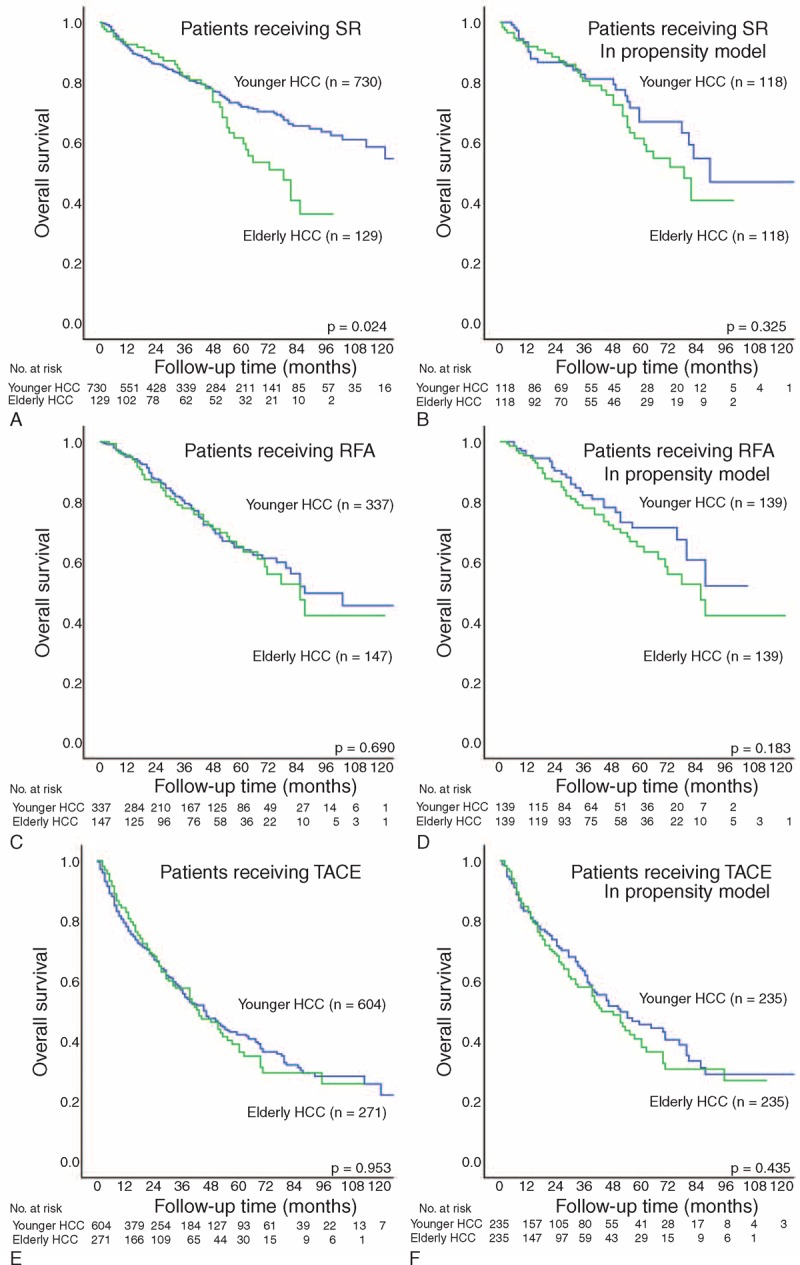
Kaplan–Meier plot with log-rank test comparing survival between elderly and younger hepatocellular carcinoma (HCC) patients receiving surgical resection (SR; panels A and B), radiofrequency ablation (RFA; panels C and D), and transarterial chemoembolization (TACE; panels E and F) in all study patients and in patients selected in the propensity model.

A total of 859 patients undergoing SR as the initial treatment were identified. Of them, 730 (85%) patients were classified as younger whereas 129 (15%) patients were categorized elderly HCC (Table 2). Elderly HCC patients had lower prevalence of hepatitis B but higher prevalence of hepatitis C (both *P* < 0.01). Elderly patients also had lower serum albumin, sodium, and AFP level and were more likely to have poorer performance status and advanced BCLC stage (all *P* < 0.05). Younger HCC patients receiving SR had significantly better survival compared with elderly HCC patients (*P* = 0.024, Figure 2A); the 1-, 3-, and 5-year estimated survival rates were 92% versus 93%, 82% versus 82%, and 72% versus 61%, respectively.

**TABLE 2:**
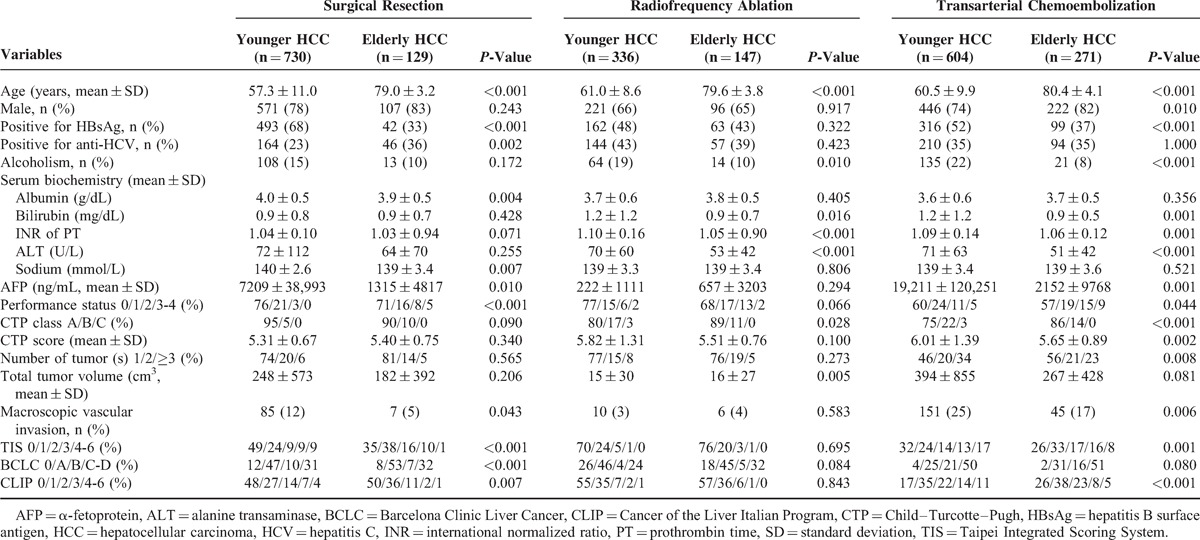
Comparison of Clinical Characteristics in Younger and Elderly Hepatocellular Carcinoma Patients Receiving Different Treatments

### Radiofrequency Ablation

A total of 483 patients receiving RFA as their primary treatment were identified. Among these patients, 336 (70%) were categorized as younger while 147 (30%) patients were elderly HCC (Table 2). Elderly HCC patients had a lower prevalence of alcoholism (*P* = 0.01), less advanced cirrhosis but larger TTV (all *P* < 0.05). There were no significant differences in sex, performance status, number of tumor(s), BCLC classification, and CLIP score. Younger and elderly HCC patients receiving RFA had similar prognosis (*P* = 0.690, Figure 2C); the 1-, 3-, and 5-year estimated survival rates were 95% versus 96%, 81% versus 78%, and 65% versus 65%, respectively.

### Transarterial Chemoembolization

A total of 875 patients received TACE as the primary therapy. Among these patients, 604 (69%) were classified as younger HCC while 271 (31%) patients were elderly (Table 2). Elderly HCC patients undergoing TACE were more likely to be male, and had a lower prevalence of hepatitis B and alcoholism (all *P* < 0.05). Elderly patients were associated with suboptimal performance status, lower serum AFP level, less advanced cirrhosis, less tumor nodule(s), and lower CLIP score (all *P* < 0.05). The long-term survival were comparable between younger and elderly patients (*P* = 0.953, Figure 2E); the 1-, 3-, and 5-year estimated survival rates were 79% versus 84%, 57% versus 57%, and 42% versus 39%, respectively.

### Patients Receiving Other Treatments

Six hundred patients (69%) receiving other treatment were classified as younger HCC while 265 (31%) patients were categorized as elderly. The long-term survival were comparable (*P* = 0.995); the 1-, 3-, and 5-year estimated survival rates were 50% versus 48%, 28% versus 31%, and 22% versus 18%, respectively.

### Propensity Score Matching Analysis

A total of 118, 139, and 235 matched pairs of patients undergoing SR, RFA, and TACE were identified in the propensity model, respectively (Table 3). There were no significant baseline differences between elderly and younger patients in the propensity model in each treatment group. Younger and elderly patients had similar long-term survival in SR, RFA, and TACE group (Figure 2B, D, and F, all *P* > 0.1).

**TABLE 3:**
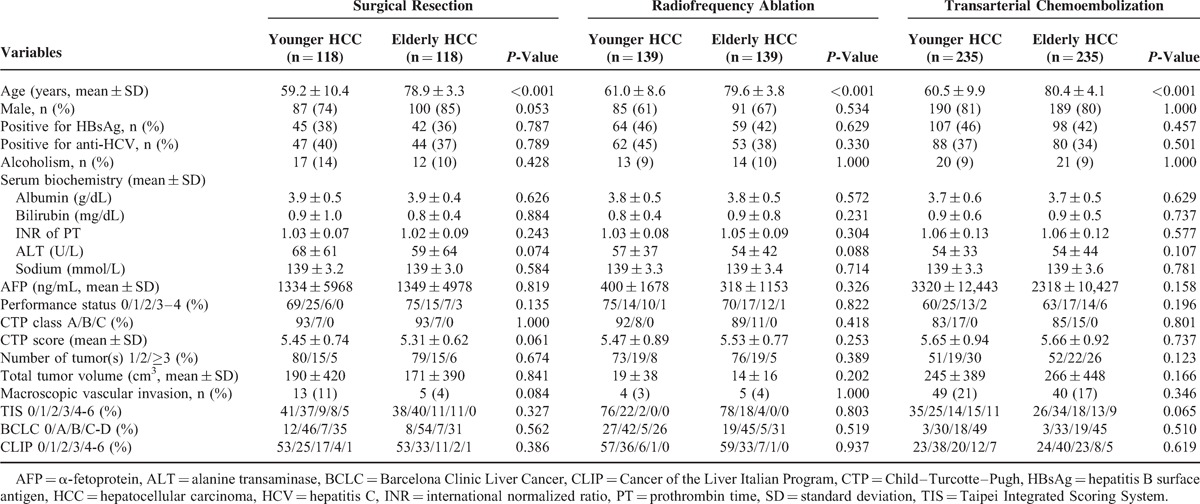
Comparison of Clinical Characteristics in Younger and Elderly Hepatocellular Carcinoma Patients Receiving Different Treatments in the Propensity Model

### Multivariate Survival Analysis

In the Cox proportional hazards model (Table 4), 7 factors were identified as independent predictors of poor prognosis in the entire cohort: AFP level ≥400 ng/dL (HR: 1.775, 95% CI: 1.551–2.032, *P* < 0.001), albumin <4.0 g/dL (HR: 1.969, 95% CI: 1.710–2.268, *P* < 0.001), bilirubin ≥1 mg/dL (HR: 1.481, 95% CI: 1.310–1.674, *P* < 0.001), multiple tumors (HR: 1.276, 95% CI: 1.133–1.436, *P* < 0.001), suboptimal performance status (HR: 1.495, 95% CI: 1.418–1.576, *P* < 0.001), TVV ≥50 cm^3^ (HR: 1.649, 95% CI: 1.436–1.892, *P* < 0.001) and macroscopic vascular invasion (HR: 2.163, 95% CI: 1.868–2.504, *P* < 0.001). For the entire population and for subgroups of patients receiving SR, RFA, or TACE, age was not an independent predictor of poor prognosis.

**TABLE 4:**
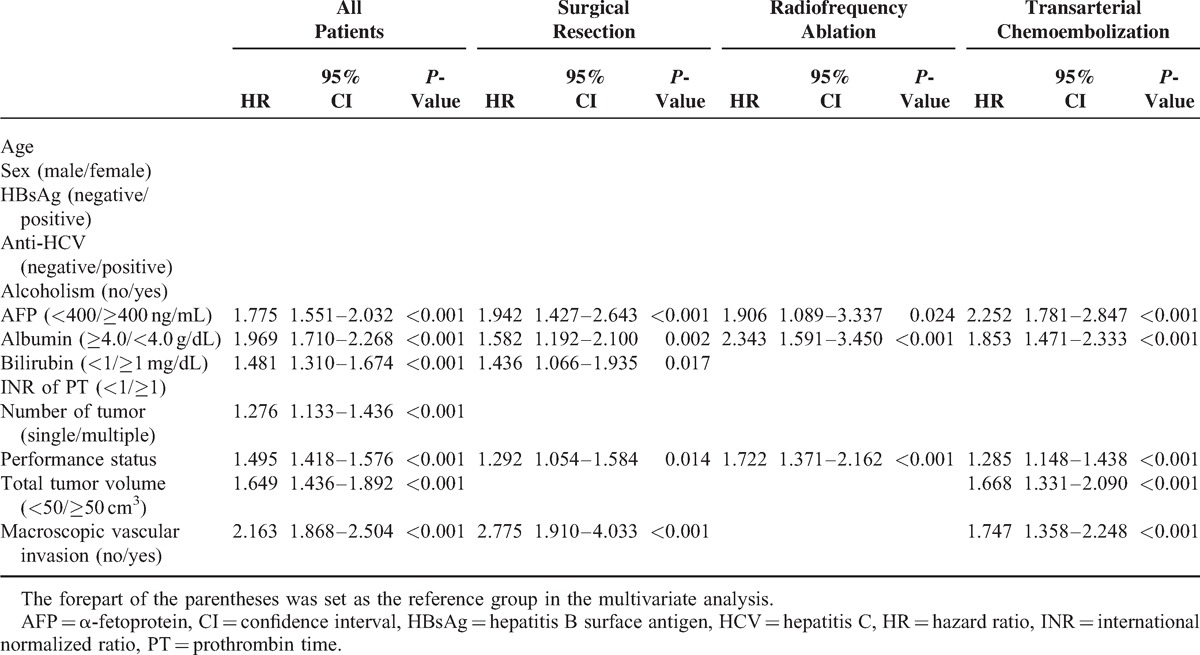
Multivariate Survival Analysis in Entire Cohort and in Patients Receiving Different Treatments

### Cancer Staging for Elderly HCC Patients

**FIGURE 3:**
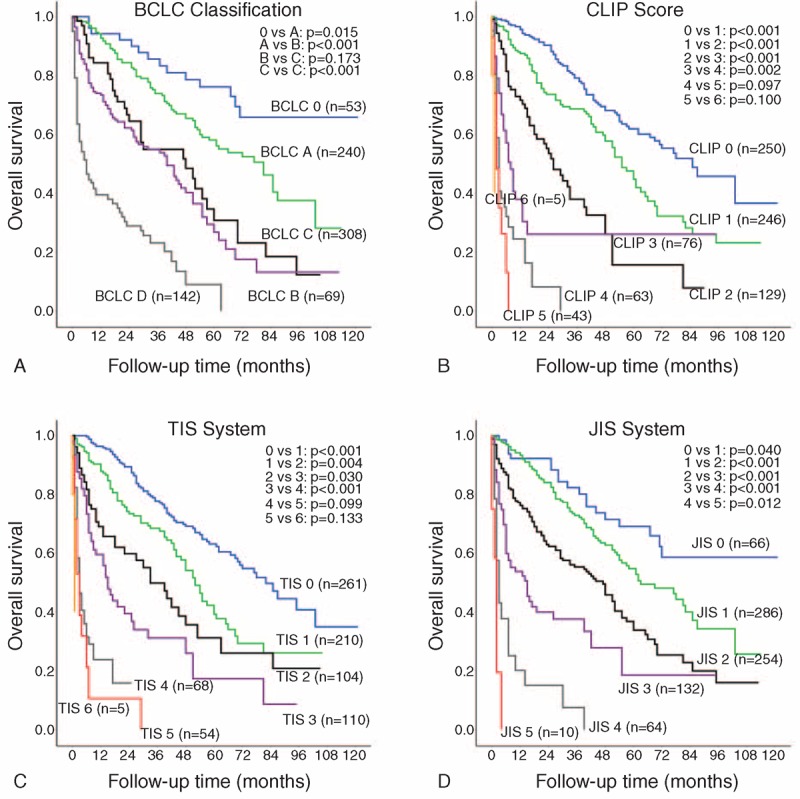
Pairwise comparison of survival between different stages of the Barcelona Clinic Liver Cancer (BCLC; panel A), Cancer of the Liver Italian Program (CLIP; panel B), Taipei Integrated Scoring System (TIS; panel C), and Japan Integrated Scoring System (JIS; panel D) in the elderly patients.

The survival distributions among elderly patients with different stages of 4 commonly used staging systems are shown in Figure 3. The staging systems were validated with both homogeneity (likelihood ratio χ^2^) and AIC method (Table 5). The CLIP score had the lowest AIC value, followed by the TIS, JIS, and lastly, BCLC system. Consistently, the CLIP system provided the highest homogeneity among the 4 staging models, followed by the TIS, JIS, and BCLC system.

**TABLE 5:**
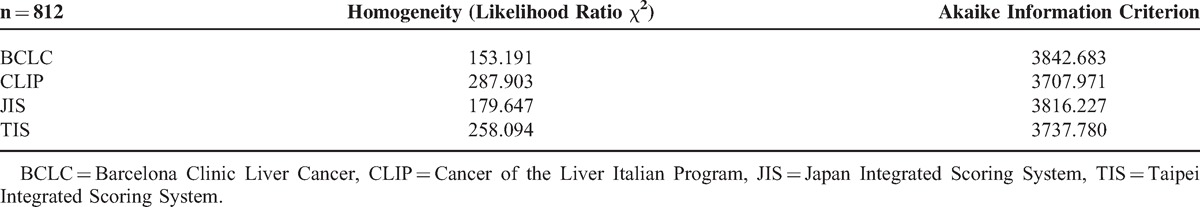
Comparison of Prognostic Ability of 4 Staging Systems Among Elderly HCC Patients

## DISCUSSION

There are growing evidence and debates on clinical features and treatment outcomes in the elderly population of HCC. We investigated a large cohort of HCC patients to clarify the impact of age on treatment allocation and long-term survival. Patients with younger and elderly HCC had diverse baseline demographics and received different treatments. With propensity score matching analysis, matched pairs of patients were generated to compare the long-term outcome in each treatment group. We demonstrated that advanced age was not associated with inferior long-term survival for HCC patients receiving SR, RFA, and TACE. These findings support the concept that aggressive treatment should be performed based on cancer stage and general condition irrespective of advanced age.

Elderly HCC patients had unique clinical presentations. We found that more elderly patients had hepatitis C, whereas more younger patients had hepatitis B or alcoholism. In the Asia-Pacific region, most HBV infection is acquired by vertical transmission at birth, while HCV infection is acquired later in life.^15^ Contrary to previous report, elderly patients in this study were more frequently associated with symptomatic HCC and had more advanced BCLC staging.^6^ However, elderly HCC patients were less often associated with advanced tumor factors, including lower serum AFP level, lower CLIP score, smaller number of tumor(s), fewer vascular invasion, and smaller TTV. The discrepancy is possibly because performance status is incorporated as part of BCLC system, therefore more symptomatic patients would be classified in advanced BCLC stage in elderly patients. Consistently, our results suggest that younger patients may have more advanced HCC than the elderly.^33^

Nearly 74% of younger patients received curative treatment or TACE, whereas 67% of elderly patients did so. The percentage of elderly patients receiving SR, RFA, or TACE increased steadily over past decades.^6,9,34^ Nevertheless, treatment strategies were different between 2 age groups in this study. Despite the progress in management of HCC, elderly patients had significantly impaired long-term survival compared with younger HCC patients in the SR group. In a retrospective study, the overall survival and surgery-related complications were similar between younger and elderly patients.^35^ In other report, advanced age was an adverse predictor of survival.^36^ It should be noticed that patients who received SR were highly selected, and 2 patient groups had discrete prognostic factors. Notably, in the propensity model, we found that age was not an independent predictor of poor prognosis. Our results suggest that SR provided comparable results for patients with elderly HCC. Advanced age should not be considered a contraindication for SR in HCC patients.

RFA is generally considered a curative treatment with predictable antitumor effect.^37^ Previous study suggested that RFA was a safe procedure in elderly patients.^38^ The less invasiveness associated with RFA renders it an acceptable alternative for patients ineligible for surgery. However, conflicting evidence existed on the long-term survival of RFA in elderly patients.^13,38^ In our analysis, more elderly HCC patients received RFA compared with younger HCC patients; this is likely because that in general, elderly patients are considered high-risk for SR.^16^ Importantly, the long-term survival was comparable in all patients and in patients selected in the propensity model. RFA thus remains a plausible option with uncompromised long-term results for elderly patients not suitable for SR.

TACE is the recommended palliative treatment for unresectable HCC.^39^ Although advanced age was once considered a contraindication for TACE, recent evidence suggested that TACE had comparable efficacy and tolerability in the elderly patients.^40,41^ Our results indicate that advanced age was not associated with decreased long-term survival, therefore TACE should be offered to elderly patients with unresectable HCC.

Consistent with published data from Japan, our results show that the 5-year overall survival rate for elderly HCC patients was around 40%.^5,15,18^ Previous reports revealed that elderly HCC patients represented roughly 15% of the entire cohort.^5,15^ However, in the current study, more than one-quarter of patients of the entire cohort were aged ≥75 years. The discrepancy could be partly explained by the rapidly aging society and our hospital being a tertiary referral center in Taiwan. Nevertheless, this study represents the largest cohort of elderly HCC ever published, and offers solid evidence on the epidemiology and clinical outcomes for elderly HCC patients.

This study recruited a large patient cohort by using a propensity model and offers convincing evidence that age does not affect the outcomes of HCC patients receiving SR, RFA, or TACE. However, there are certain limitations. Firstly, the retrospective nature makes this study vulnerable to potential bias. Even with careful propensity score matching analysis with a pre-defined caliper, these biases might not be completely avoided. Secondly, this single-center study was performed in the Asia-Pacific region, a highly hepatitis B endemic area, and external validation is needed from different study groups.

In conclusion, elderly HCC patients have discrete clinical characteristics and less often undergo aggressive treatment compared with younger patients. With the advances in treatment strategy, elderly HCC patients receiving SR, RFA, and TACE share similar long-term prognosis with younger HCC patients. Elderly patients should be encouraged for active anti-cancer treatment based on cancer stage and general condition, irrespective of the advanced age. The CLIP system can be used as a prognostic model to assess the outcome in this special group of patients.
