# Uncompromised Treatment Efficacy in Elderly Patients With Hepatocellular Carcinoma: A Propensity Score Analysis: Erratum

**DOI:** 10.1097/01.md.0000464208.55673.5b

**Published:** 2015-01-30

In the article “Uncompromised Treatment Efficacy in Elderly Patients With Hepatocellular Carcinoma: A Propensity Score Analysis”,^1^ which appeared in Volume 93, Issue 28 of *Medicine*, one of the authors’ affiliations, National Yang-Ming University School of Medicine, Taipei, Taiwan, was omitted. The article has since been corrected online.
